# Sleep quality in women with diabetes in pregnancy: a single-center retrospective study

**DOI:** 10.1186/s12884-023-05905-x

**Published:** 2023-08-22

**Authors:** Qizhen Wu, Zhaoyan Meng, Qing Liu, Lili Zhang, Baohong Mao, Cheng Wang, Min Zhou, Zhi Zhang, Dehua Yang, Ruirui Jin, Tongying Yi

**Affiliations:** Gansu Provincial Maternity and Child-Care Hospital, No. 143 North Street, Qilihe District, 730050 Lanzhou City, Gansu Province China

**Keywords:** Diabetes in pregnancy, Sleep quality, Pittsburg Sleep Quality Index, STOP-BANG questionnaire

## Abstract

**Purpose:**

Sleep quality is an important indicator of individual quality of life, which not only affects people’s mental health but is also closely related to the occurrence of many diseases. Sleep disorders associated with diabetes in pregnancy can greatly endanger the health of both mothers and babies, and their hazards are strongly associated with blood glucose levels. This study explored the quality of sleep and sleep disorders in pregnant women with diabetes.

**Methods:**

From June 2020 to July 2021, a total of 693 patients diagnosed with diabetes during pregnancy in Gansu Provincial Maternal and Child Health Hospital were used as the experiment group, including 626 patients with gestational diabetes mellitus (GDM) and 67 patients with pregestational diabetes mellitus (PGDM). At the same time, 709 women not having diabetes were randomly selected as the control group. To obtain the general situation of the participants, the participants were surveyed using the Pittsburgh Sleep Quality Index (PSQI) and the STOP-BANG (S, Snoring; T, Tiredness; O, Observed apnea; P, high blood Pressure; B, Body mass index > 35 kg/ m^2^; A, Age > 50 years; N, Neck circumference > 40 cm; G, male Gender) questionnaire. The differences in sleep quality and obstructive sleep apnea-hypopnea syndrome (OSAHS) were analyzed between the experiment group and the control group by using chi-square and t-test, and the clinical features and related factors of sleep disorder were analyzed.

**Results:**

Compared with the control group, the age, pre-pregnancy weight, body mass index (BMI), and neck circumference were larger in the experimental group (P < 0.05). The experimental group had higher PSQI scores for sleep quality, time to fall asleep score, sleep duration, sleep efficiency, sleep disorder, and daytime dysfunction than the control group (all P < 0.001). Specific analysis of the clinical features of sleep disorders indicated that the experimental group scored higher than the control group (P < 0.05). The analysis of the types of daytime dysfunction showed that the experiment group scored higher in terms of frequently feeling sleepy and lack of energy to do things than the control group (P < 0.05). Analysis of STOP-BANG scores indicated that the proportion of patients with GDM or PGDM having fatigue, hypertension, BMI > 35 kg/m^2^, and neck circumference > 40 cm was higher than that in the control group (P < 0.05). According to regression analysis, sleep quality of patients with GDM was significantly impacted by the increases in age (OR: 1.243, CI:1.197–1.290), neck circumference (OR: 1.350, CI: 1.234–1.476), PSQI score (OR: 2.124, CI:1.656–2.724), and sleep efficiency score (OR: 3.083, CI:1.534–6.195), whereas that of patients with PGDM was impacted by age (OR: 1.191, CI:1.086–1.305), neck circumference (OR: 1.981, CI: 1.469–2.673), and PSQI score (OR: 7.835, CI: 2.383–25.761).

**Conclusions:**

Pregnant women with diabetes had poorer sleep quality and a higher risk of developing OSAHS than those without diabetes. There may be some link between sleep quality and the onset of diabetic.

## Introduction

Diabetes in pregnancy includes pregestational diabetes mellitus (PGDM) and gestational diabetes mellitus (GDM). PGDM may be diagnosed before or during pregnancy. GDM is characterized by the abnormality in glucose metabolism to varying degrees that occurs or is first detected during pregnancy. In recent years, improvements in living standards and changes in lifestyle have resulted in the increased annual incidence of GDM and PGDM in China, with an incidence rate of more than 10%. Diabetes in pregnancy can increase the incidence of preterm birth, hypertension, infection, and diseases of the cardiovascular system and can increase the risk of metabolic diseases such as congenital malformations, hypoglycemia, sleep disturbances, and polycythemia in new-borns [1–3]. Sleep is an indispensable part of people’s lives, and every individual spends 1/3rd of their life in sleep. Previous studies have reported that circadian rhythm disturbances caused by various types of sleep disorders can impair glucose metabolism by affecting insulin sensitivity and β-cell function. Sleep disturbances have been reported to increase the risk of hypertension, obesity, dyslipidemia, depression, and dementia [4–7]. The sleep quality of pregnant women with diabetes is easily neglected, but sleep quality seriously affects the quality of life of pregnant women. Short sleep time is associated with an increased risk of diabetes, cardiovascular disease, coronary heart disease, and obesity [8]. A study found that high-risk (gestational diabetes and hypertension) pregnant women had lower sleep quality and moderate quality of life [9]. A clinical study in China found that poor sleep quality and shortened sleep duration during pregnancy in turn increased the risk of gestational diabetes [10]. Recent studies have reported that sleeping for too long or too short time may increase the risk of mortality in people with diabetes, especially in those diagnosed with diabetes at a young age and those with severe diabetes who receive both insulin and oral hypoglycemic drugs [11]. Although the association between hyperglycemia and sleep duration is not fully understood, it is generally accepted that sleeping for too short time may reduce insulin sensitivity, leading to insulin resistance and obesity, which in turn leads to increased blood sugar [12]. Longer sleep duration may be related to low income levels and poor physical conditions [13]. According to a recent study, bedtime at night, night-time sleep time, and daytime nap times were associated with increased blood glucose, especially in women [14].

Therefore, the sleep quality of pregnant women with diabetes requires our attention. However, in China, only a few studies have analyzed the sleep quality of patients with GDM and PGDM and the risk of several sleep disorders. The purpose of this study was to analyze sleep quality and the occurrence of sleep disorders in women with diabetes in pregnancy.

## Methods

### Study design and participants

From June 2020 to July 2021, 693 patients diagnosed with diabetes in pregnancy (626 patients with GDM and 67 patients with PGDM) in Gansu Provincial Maternity and Child-Care Hospital were considered the experimental group. At the same time, 709 pregnant women without diabetes were randomly selected as the control group.

PGDM was confirmed if either of the following two criteria was met [15, 16]: (1) Patients diagnosed with diabetes before pregnancy and (2) Pregnant women who had not undergone blood glucose examination before pregnancy, especially those with high-risk factors for diabetes, needed to clarify whether diabetes was present at the first prenatal examination. PGDM was diagnosed if any of the following criteria was met during pregnancy: (1) fasting plasma glucose (FPG) ≥ 7.0 mmol/L (126 mg/dL); (2) blood glucose 2 h after taking 75 g sugar in the oral glucose tolerance test (OGTT) ≥ 11.1 mmol/L (200 mg/dL), with typical hyperglycemia symptoms or hyperglycemic crisis, random blood glucose ≥ 11.1 mmol/L (200 mg/dL), or glycosylated hemoglobin (HbA1c) ≥ 6.5%. The national glycohemoglobin standardization program (NGSP)/diabetes control and complication trial (DCCT) was used. The risk factors included obesity (especially severe obesity), type 2 diabetes mellitus in first-degree relatives, history of GDM, macrosomia, polycystic ovary syndrome, and repeated positive fasting glucose in the first trimester of pregnancy.

For all pregnant women who had not been diagnosed with PGDM or GDM, the OGTT was performed at 24–28 weeks of gestation. The 75-g OGTT method was performed as follows. Women were told to fast for at least 8 h before the OGTT. Additionally, they were asked to stay on a normal diet 3 days prior to the test and eat no less than 150 g of carbohydrates per day. At the time of examination, 300 mL liquid containing 75 g of glucose was administered orally within 5 min. Then, venous blood of pregnant women was drawn before and 1 and 2 h after administering sugar (calculating the time from the beginning of drinking glucose water). The glucose oxidase test was used to determine blood glucose levels after placing a test tube containing sodium fluoride. Diagnostic criteria for 75-g OGTT method were as follows [17]: Blood glucose values < 5.1, 10.0, and 8.5 mmol/L (92, 180, and 153 mg/dL) before and 1 and 2 h after taking sugar, respectively. Patients with the blood glucose level meeting or exceeding the aforementioned criteria were diagnosed as having GDM. Patients with the first detection during pregnancy and the increase in the blood glucose level reaching the criteria for diabetes mellitus were diagnosed as having PGDM rather than GDM. OGTT-negative pregnant women were included in the control group.

### Data collection and quality control

Based on the information of electronic medical record registration and medical history, we collected relevant data including the age, pre-pregnancy weight, pre-pregnancy body mass index (BMI), height, neck circumference, number of pregnancies and deliveries, comorbidities and complications before and during pregnancy, and contact information of the pregnant women. Each pregnant woman was consulted by a trained physician who reviewed the electronic medical record and medical history. Once the inclusion criteria were met, the basic information was recorded in a computer database. The participants were instructed to complete the Pittsburgh Sleep Quality Index (PSQI) scale and STOP-BANG (S, Snoring; T, Tiredness; O, Observed apnea; P, high blood Pressure; B, Body mass index > 35 kg/ m2; A, Age > 50 years; N, Neck circumference > 40 cm; G, male Gender) questionnaire at 24–28 weeks of uniform gestational age. To ensure the accuracy of the research, samples and information were collected and maintained by special personnel. A double-blind entry rule was adopted for data entry. Parallel entry was performed by two listed persons.

### Sleep quality assessment

PSQI was used to measure sleep quality in the last month and to distinguish between good and poor sleep quality [18]. It included 19 questions categorized into seven sleep components: (1) subjective sleep quality (1 item); (2) sleep latency (2 items); (3) night sleep time (1 item); (4) sleep efficiency (3 items); (5) sleep disorders (9 items); (6) use of sleep drugs (1 item); and (7) daytime dysfunction (2 items). The score for each group ranged from 0 to 3, where 3 represented the greatest dysfunction. The sleep score added up to a total score of 0–21. The higher the total score, the worse was the quality of sleep, with 0–5, > 5 points indicating good, and poor sleep qualities, respectively. The PSQI score of > 5 was considered to denote poor sleep quality, with a sensitivity of 89.6%, specificity of 86.5%, and an internal consistency of 0.83 for Cronbach’s alpha [18]. The Chinese version of PSQI had good overall reliability (R = 0.82–0.83) and repeatability (R = 0.77–0.85) [19]. The PSQI had been validated for assessing the quality of perinatal sleep, supporting its use during pregnancy [20, 21].

### Assessment of the risk of obstructive sleep apnea-hypopnea syndrome development

The STOP-BANG questionnaire consists of eight acronyms [22]. The patient answered the first four questions (STOP questions). The researchers measured the patients’ height, weight, blood pressure, and neck circumference and then answered the last four questions (BANG questions). The answer was yes (1 point) or no (0 points). The total score was calculated, which was found to range from 0 to 8. The higher the score, the greater was the risk. The score of ≥ 3 or < 3 was considered as high-risk or low-risk OSAHS, respectively.

### Statistical analysis

The data were statistically analyzed using SPSS version 22.0 (SPSS, Inc., Chicago, IL, USA). The continuous variables that conformed to a normal distribution are expressed as the mean ± SD. The continuous variables that did not conform to a normal distribution are expressed as median (range). The count data are expressed as n (%). The normal metrology data are expressed as $\stackrel{-}{\text{x} }$± S. The quantitative comparison was performed using the Student’s t test, whereas the rate comparison was performed the using chi-square test. Linear regression analysis of sleep quality of the GDM or PGDM group was performed using the Wald test. A P value of < 0.05 was considered statistically significant.

## Results

### Participant characteristics

A total of 693 patients with diabetes in pregnancy, including 626 patients with GDM and 67 patients with PGDM, were considered as the experiment group. Overall, 709 non-diabetic pregnant women were included in the control group. The results showed that age, preconception weight, preconception BMI, and neck circumference between the diabetes in pregnancy group and the non-diabetic control group were significantly different (all P < 0.001). However, there was no significant difference in the height between the two groups (Table 1).

### PSQI scores

Compared with the control group, patients with GDM or PGDM had high overall PSQI scores, higher proportion of patients with a score of > 5 and worse sleep quality (P < 0.001; Table 2). The patients with GDM or PGDM exhibited higher PSQI scores for sleep quality, time to fall asleep score, sleep duration, sleep efficiency, sleep disorder, and daytime dysfunction (P < 0.05) than those in the control group (all P < 0.001). There is no need to compare the two groups because none of the participants were taking hypnotic medications, which were not shown in Table 2.

### Sleep disorder analysis

The study compared the risk of sleep disorders between the two groups of patients and found that the scores of difficulties falling asleep, ease of waking up at night or waking up early, poor breathing, cough or loud snoring, feeling hot, nightmares, pain, feeling cold, and other events that affected sleep of pregnant women with diabetes (including GDM and PGDM) were significantly higher than those of controls (all P < 0.05). In terms of going to the toilet at night, the score of the GDM group was significantly lower than that of the control group (P < 0.001); However, it did not differ significantly between the PGDM group and the control group (P = 0.067) (Table 3).

### Analysis of daytime dysfunction and STOP-BANG scores

After analyzing the types of daytime dysfunction, it was observed that patients with GDM or PGDM scored higher in terms of frequently feeling sleepy and lack of energy to do things than the patients in the control group (P < 0.05; Table 4).

Patients with GDM or PGDM exhibited higher overall STOP-BANG scores than patients in the control group (P < 0.05; Tables 5 and 6). The comparison of age and gender in STOP-BANG scores was removed since all participants were pregnant women under the age of 50. Analysis of STOP-BANG scores indicated that the proportion of patients with GDM or PGDM having fatigue, hypertension, BMI > 35 kg/m^2^, and neck circumference > 40 cm was higher than that of patients in the control group (P < 0.05); however, the GDM or PGDM group and the control group patients exhibited no significant difference in terms of snore and breathing stops.

### Multifactorial analysis of sleep quality

The selection of variable for inclusion in the regression analysis for sleep quality on GDM and PGDM was made possible by the unifactorial comparison of the population characteristics and all the scores. Sleep quality of the patients with GDM was significantly impacted by the increase in age (OR: 1.243, CI: 1.197–1.290), neck circumference (OR: 1.350, CI: 1.234–1.476), PSQI score (OR: 2.124, CI: 1.656–2.724), and sleep efficiency score (OR: 3.083, CI:1.534–6.195) (P < 0.05; Table 7). With the exception of the sleep efficiency score, the same variables were found to affect sleep quality of the PGDM group (age, OR: 1.191, CI: 1.086–1.305; neck circumference, OR: 1.981, CI: 1.469–2.673; PSQI score, OR: 7.835, CI: 2.383–25.761) (P < 0.05; Table 8).

Thus, we obtained the following regression equations:

Sleep quality on GDM = 0.217 * age + 0.300 * neck circumference + 0.753 * PSQI score + 1.126 * sleep efficiency score – 17.272.

Sleep quality on PGDM = 0.175 * age + 0.684 * neck circumference + 2.059 * PSQI score.

Intriguingly, STOP-BANG was left out of the regression equation in both groups despite significant differences in univariate analysis.

**Table 1:** Characteristics of study population

	The control group	Diabetes in pregnancy		t_1_ (GDM vs. controls)	*P*_1_	t_2_ (PGDM vs. controls)	*P*_2_
	(n = 709)	GDM (n = 626)	PGDM (n = 67)				
Age (years)	28.27 ± 3.56	31.73 ± 4.41	31.39 ± 3.93	15.659	< 0.001	6.805	< 0.001
Prepregnancy weight (kg)	54.94 ± 8.45	62.07 ± 15.67	67.28 ± 10.71	10.157	< 0.001	9.163	< 0.001
Height (cm)	161.12 ± 4.89	161.44 ± 4.91	160.88 ± 4.54	1.177	0.239	-0.387	0.682
Prepregnancy BMI (kg/m^2^)	21.14 ± 3.10	23.59 ± 3.83	25.58 ± 3.65	12.753	< 0.001	11.047	< 0.001
Neck circumference (cm)	33.13 ± 1.74	34.63 ± 2.25	35.98 ± 2.63	13.464	< 0.001	8.679	< 0.001

Abbreviations: GDM, gestational diabetes mellitus; PGDM, pregestational diabetes mellitus; BMI, body mass index

**Table 2:** Comparison of PSQI scores

	The control group	GDM	t/χ^2^ (GDM vs. control)	P _1_value	PGDM	t/χ^2^ (PGDM vs. control)	*P*_2_ value
	n(%)	n(%)			n(%)		
PSQI Score	3.57 ± 1.71	5.74 ± 2.81	16.748	< 0.001*	6.24 ± 3.12	6.903	< 0.001*
PSQI grade			189.194	< 0.001*		69.830	< 0.001*
0–5 points	623(87.9)	338(54.0)			33(49.3)		
>5 points	86(12.1)	288(46.0)			34(50.7)		
Sleep quality score			66.871	< 0.001*		64.756	< 0.001*
0	175(67.05)	76(29.12)			10(3.83)		
1	441(51.34)	384(44.70)			34(3.96)		
2	93(35.50)	151(57.63)			18(6.87)		
3	0(0.00)	15(75.00)			5(25.00)		
Time to fall asleep score			87.222	< 0.001*		37.580	< 0.001*
0	175(51.78)	145(42.90)			18(5.33)		
1	432(59.02)	270(36.89)			30(4.10)		
2	98(35.38)	166(59.93)			13(4.69)		
3	4(7.27)	45(81.82)			6(10.91)		
Sleep duration score			186.122	< 0.001*		151.201	< 0.001*
0	681(59.58)	425(37.18)			37(3.24)		
1	24(10.96)	169(77.17)			26(11.87)		
2	4(13.33)	23(76.67)			3(10.00)		
3	0(0.00)	9(90.00)			1(10.00)		
Sleep efficiency score			270.433	< 0.001*		285.797	< 0.001*
0	705(60.78)	416(35.86)			39(3.36)		
1	0(0.00)	137(88.39)			18(11.61)		
2	0(0.00)	36(83.72)			7(16.28)		
3	4(9.09)	37(84.09)			3(6.82)		
Sleep disorder score			89.897	< 0.001*		31.937	< 0.001*
0	16(38.10)	23(54.76)			3(7.14)		
1	638(56.81)	438(39.00)			47(4.19)		
2	55(23.81)	160(69.26)			16(6.93)		
3	0(0.00)	5(83.33)			1(16.67)		
Daytime dysfunction score			163.281	< 0.001*		53.786	< 0.001*
0	383(74.08)	128(24.76)			6(1.16)		
1	158(35.51)	256(57.53)			31(6.97)		
2	138(40.95)	178(52.82)			21(6.23)		
3	30(29.13)	64(62.14)			9(8.74)		

Abbreviations: PQSI, Pittsburgh Sleep Quality Index; GDM, gestational diabetes mellitus; PGDM, pregestational diabetes mellitus;

**Table 3:** Classification comparison of sleep disorders

	The control group	GDM	χ^2^	*P value*	PGDM	χ^2^	*P value*
	n(%)	n(%)			n(%)		
Difficulty falling asleep			38.458	< 0.001*		11.715	0.008*
0	425(56.97)	289(38.74)			32(4.29)		
1	107(48.64)	106(48.18)			7(3.18)		
2	77(33.33)	138(59.74)			16(6.93)		
3	100(48.78)	93(45.37)			12(5.85)		
Easy to wake up at night or wake up early			91.705	< 0.001*		29.722	< 0.001*
0	379(65.01)	189(32.42)			15(2.57)		
1	121(51.49)	100(42.55)			14(5.96)		
2	130(37.79)	195(56.69)			19(5.52)		
3	79(32.92)	142(59.17)			19(7.92)		
Go to the toilet at night			11.519	< 0.001*		7.168	0.067
0	63(46.67)	64(47.41)			8(5.93)		
1	51(39.53)	70(54.26)			8(6.20)		
2	213(47.97)	205(46.17)			26(5.86)		
3	382(55.04)	287(41.35)			25(3.60)		
Poor breathing			49.157	< 0.001*		22.739	< 0.001*
0	615(55.11)	455(40.77)			46(4.12)		
1	57(40.43)	73(51.77)			11(7.80)		
2	23(22.77)	69(68.32)			9(8.91)		
3	14(31.82)	29(65.91)			1(2.27)		
Cough or snoring high			168.495	< 0.001*		143.565	< 0.001*
0	669(59.05)	423(37.33)			41(3.62)		
1	28(26.17)	75(70.09)			4(3.74)		
2	8(7.84)	80(78.43)			14(13.73)		
3	4(6.67)	48(80.00)			8(13.33)		
Feeling cold			19.704	0.001*		10.756	0.005*
0	673(51.69)	567(43.55)			62(4.76)		
1	32(47.06)	34(50.00)			2(2.94)		
2	4(14.81)	20(74.07)			3(11.11)		
3	0(0.00)	5(100.00)			0(0.00)		
Feeling hot			99.107	< 0.001*		45.975	< 0.001*
0	547(60.85)	322(35.82)			30(3.34)		
1	44(37.61)	68(58.12)			5(4.27)		
2	72(33.49)	129(60.00)			14(6.51)		
3	46(26.90)	107(62.57)			18(10.53)		
Nightmares			33.559	< 0.001*		17.823	< 0.001*
0	551(55.60)	401(40.46)			39(3.94)		
1	99(40.57)	132(54.10)			13(5.33)		
2	51(38.35)	70(52.63)			12(9.02)		
3	8(23.53)	23(67.65)			3(8.82)		
Pain			57.844	< 0.001*		19.383	< 0.001*
0	628(55.43)	458(40.42)			47(4.15)		
1	51(36.69)	77(55.40)			11(7.91)		
2	22(23.91)	63(68.48)			7(7.61)		
3	8(21.05)	28(73.68)			2(5.26)		
Others			84.974	< 0.001*		51.430	< 0.001*
0	670(55.37)	491(40.58)			49(4.05)		
1	31(29.52)	64(60.95)			10(9.52)		
2	4(6.78)	50(84.75)			5(8.47)		
3	4(14.29)	21(75.00)			3(10.71)		

Abbreviations: GDM, gestational diabetes mellitus; PGDM, pregestational diabetes mellitus

**Table 4:** Classification comparison of daytime dysfunction

	The control group	GDM	χ^2^	*P value*	PGDM	χ^2^	*P value*
	n(%)	n(%)			n(%)		
Felt sleepy			126.968	< 0.001		37.977	< 0.001
0	453(65.18)	222(31.94)			20(2.88)		
1	110(41.51)	142(53.58)			13(4.91)		
2	77(26.64)	191(66.09)			21(7.27)		
3	69(45.10)	71(46.40)			13(8.50)		
Lack of energy to do things			135.229	< 0.001		62.061	< 0.001
0	431(68.09)	188(29.70)			14(2.21)		
1	223(39.61)	308(54.71)			32(5.68)		
2	35(24.65)	90(63.38)			17(11.97)		
3	20(31.25)	40(62.50)			4(6.25)		

Abbreviations: GDM, gestational diabetes mellitus; PGDM, pregestational diabetes mellitus

**Table 5:** Comparison of STOP-Bang scores

	The control group	Diabetes in pregnancy		t_1_ (GDM vs. controls)	P_1_	t_2_ (PGDM vs. controls)	P_2_
	**(n = 709)**	**GDM (n = 626)**	**PGDM (n = 67)**				
**Score**	0.50 ± 0.73	0.77 ± 0.77	1.09 ± 0.81	6.425	< 0.001	6.175	< 0.001

Abbreviations: GDM, gestational diabetes mellitus; PGDM, pregestational diabetes mellitus

**Table 6:** STOP-Bang score items

	The control group	GDM	χ^2^	*P value*	PGDM	χ^2^	*P value*
	n(%)	n(%)			n(%)		
Snore			0.002	0.991		3.343	0.068
No	565(50.86)	499(44.91)			47(4.23)		
Yes	144(49.48)	127(43.64)			20(6.87)		
Fatigue			106.925	< 0.001		46.596	< 0.001
No	547(61.74)	313(35.33)			26(2.93)		
Yes	162(31.40)	313(60.66)			41(7.95)		
Stop breathing			0.253	0.615		0.071	0.790
No	701(50.65)	617(44.58)			66(4.77)		
Yes	8(44.44)	9(50.00)			1(5.56)		
Hypertension			13.033	< 0.001		33.883	< 0.001
No	705(51.38)	606(44.17)			61(4.45)		
Yes	4(13.33)	20(66.67)			6(20.00)		
BMI > 35 kg/ m^2^			5.684	0.017		21.219	< 0.001*
No	709(50.82)	621(44.52)			65(4.66)		
Yes	0(0.00)	5(71.43)			2(28.57)		
Neck circumference > 40 cm			5.684	0.017		31.869	< 0.001*
No	709(50.86)	621(44.55)			64(4.59)		
Yes	0(0.00)	5(62.50)			3(37.50)		

Abbreviations: GDM, gestational diabetes mellitus; PGDM, pregestational diabetes mellitus; BMI, body mass index. *, Fisher’s precision probability test

**Table 7:** Multifactorial effect of sleep quality on gestational diabetes mellitus

variate	β	SE	Wald χ^2^	OR(95%CI)	*P value*
Age (years)	0.217	0.019	131.808	1.243(1.197 ~ 1.290)	<0.001
Prepregnancy weight (kg)	0.014	0.022	0.383	1.014 (0.970 ~ 1.059)	0.536
Height (cm)	-0.026	0.022	1.383	0.975(0.934 ~ 1.017)	0.240
Prepregnancy BMI (kg/m^2^)	0.085	0.063	1.789	1.088(0.961 ~ 1.232	0.181
Neck circumference (cm)	0.300	0.046	42.822	1.350(1.234 ~ 1.476)	<0.001
PSQI Score	0.753	0.127	35.195	2.124(1.656 ~ 2.724)	<0.001
Sleep quality score	-0.107	0.106	1.006	0.899(0.730 ~ 1.107)	0.316
Time to fall asleep score	0.141	0.095	2.201	1.152(0.956 ~ 1.387)	0.138
Sleep duration score	-0.467	0.378	1.530	0.627(0.299 ~ 1.314)	0.216
Sleep efficiency score	1.126	0.356	10.003	3.083(1.534 ~ 6.195)	0.002
Sleep disorder score	0.221	0.119	3.433	1.247(0.987 ~ 1.576)	0.064
Daytime dysfunction score	0.084	0.089	0.890	1.087(0.914 ~ 1.294)	0.346
STOP-Bang score	-0.102	0.542	0.036	903(0.312 ~ 2.611)	0.850
constant	-17.272	3.670	22.152		<0.001

Abbreviation: SE, standard error; OR, odds ratio; BMI, body mass index

**Table 8:** Multifactorial effect of sleep quality on pregestational diabetes mellitus

variate	β	SE	Wald χ^2^	OR(95%CI)	*P value*
Age (years)	0.175	0.047	13.836	1.191(1.086 ~ 1.305)	<0.001
Prepregnancy weight (kg)	0.143	0.24	1.320	1.153(0.904 ~ 1.471)	0.251
Height (cm)	-0.143	0.103	1.933	0.866(0.708 ~ 1.060)	0.164
Prepregnancy BMI (kg/m^2^)	-0.255	0.322	0.626	0.775(0.413 ~ 1.457)	0.429
Neck circumference (cm)	0.684	0.153	20.023	1.981(1.469 ~ 2.673)	<0.001
PSQI Score	2.059	0.607	11.489	7.835(2.383 ~ 25.761)	0.001
Sleep quality score	0.049	0.495	0.010	1.050(0.398 ~ 2.770)	0.922
Time to fall asleep score	-0.263	0.487	0.293	0.768(0.296 ~ 1.995)	0.588
Sleep duration score	0.712	1.140	0.390	2.038(0.218 ~ 19.051)	0.533
Sleep efficiency score	1.573	0.981	2.572	4.821(0.705 ~ 32.962)	0.109
Sleep disorder score	-0.442	0.550	0.645	0.643(0.219 ~ 1.889)	0.422
Daytime dysfunction score	0.029	0.498	0.003	1.030(0.388 ~ 2.733)	0.953
STOP-Bang score	-0.063	0.551	0.013	0.939(0.319 ~ 2.761)	0.909
constant	-11.182	16.786	0.444		0.505

Abbreviation: SE, standard error; OR, odds ratio; BMI, body mass index

## Discussion

Sleep quality during pregnancy has a profound effect on health of both the mother and the child. Some studies have reported that sleep disturbances during pregnancy may be associated with pregnancy-induced hypertension syndrome, preterm birth, increased cesarean section rates [23–26], and an increased risk of attention deficit hyperactivity disorder in children [27]. Improving sleep quality in pregnant women with diabetes may be beneficial for improving glycemic control and pregnancy outcomes. In this series, we sought to assess the sleep quality and the risk of OSAHS in patients with PGDM and GDM compared with those in patients with non-gestational diabetes. Our results showed that pregnant women with diabetics had poorer sleep quality, more sleep disturbances, and a higher risk of developing OSAHS than pregnant women without diabetes. This result is consistent with those of previous studies [9, 28].

The results of this study demonstrated that patients with diabetes in pregnancy were older and had a larger neck circumference and higher preconception weight and preconception BMI than patients in the control group. This finding is consistent with the risk factors for diabetes in pregnancy reported in previous studies [29, 30].

By comparing the PSQI scores between pregnant women with non-gestational diabetes and non-diabetic controls, we found that the patients with GDM or PGDM had higher overall PSQI scores than the control group patients. The findings indicated that sleep quality in pregnant patients with diabetes was poor. Compared with the control group, pregnant patients with diabetes had shorter sleep time, less efficient sleep, and more sleep disorders and daytime dysfunction.

After analyzing the specific types of sleep disturbances in patients, we observed that patients with GDM or PGDM scored higher than the control group in terms of many events that affect sleep. Additionally, the pregnant patients with diabetes had more sleep disturbances. In terms of going to the toilet at night, the score of the GDM group was significantly lower than that of the control group, but the score did not differ significantly between the PGDM group and the control group. This suggests that the frequency of going to the toilet at night is low in pregnant women with diabetes. This might be due to the frequent urination at night, which is not uncommon in pregnant women and pregnant women with diabetes.

All of these have a big impact on sleep quality. However, because the main factor affecting sleep quality of pregnant women without diabetics is going to the toilet at night, these people subjectively believe that the score of going to the toilet at night is higher than that of other diseases that affect sleep quality. Analysis of the type of daytime dysfunction in the study participants indicated that the pregnant patients with diabetes had higher scores in terms of frequent sleepiness and lack of energy to do things than the control group patients. Whether there is a correlation between sleep disturbances and gestational diabetes remains controversial. A meta-analysis reported that sleeping for too short time increases the risk of GDM [31]. However, results from another meta-analysis suggested a significant association between longer but not shorter sleep duration and GDM incidence [32]. According to another study, the relationship between the sleep duration of patients during different trimesters and the risk of developing GDM in the first trimester was unclear [33]. Moreover, some studies have reported that a short sleep duration in the second trimester was associated with the development of GDM [34, 35]. Studies from China have reported that poor sleep quality may be associated with high blood glucose or GDM during pregnancy [36]. Therefore, to determine whether sleep is related to diabetes in pregnancy, objective indicators such as polysomnography (PSG) are needed. Moreover, future prospective studies with a large sample size are needed.

OSAHS causes intermittent hypoxemia, sleep deprivation, and hypercapnia at night, which can cause insulin resistance and affect glucose metabolism. Increasing studies have confirmed that OSAHS is strongly associated with abnormalities in glucose metabolism. Moderate-to-severe sleep breathing disorders in the first trimester of pregnancy elevated the risk of GDM compared with mild sleep disorders. In the present study, patients with GDM/PGDM had a higher risk of developing OSAHS than pregnant women without diabetes, which is consistent with the findings of aforementioned studies.

Overall, the accuracy of subjective and objective assessments of sleep during pregnancy by using the existing screening tools is limited, and these assessments are usually conducted by recording clinical data and standardizing scale questionnaires. By contrast, the STOP-BANG questionnaire is simple to operate and has high sensitivity and specificity [37]. In future studies, more appropriate screening tools should be developed for use during pregnancy, or PSG should be used to demonstrate the relationship between OSAHS and GDM.

This study has some limitations. First, this study was a single-center retrospective study. Future studies should include multiple centers to obtain more definitive results. Second, this study was conducted only in patients with diabetes in pregnancy in China, leading to selection bias, which may be due to the small sample size of the study; In addition, China is a multi-ethnic country, and pregnant women from different ethnic groups have differences in their living environment and habits, which may have an impact on sleep quality, so the patients included in the study cannot represent the whole population of pregnant women with diabetes in China. Third, this study was a kind of investigation, inevitably leading to some biases. Fourth, pregnant women with negative glucose tolerance tests at 24–28 weeks of gestation constituted the control group in this study; however, the possibility of abnormal blood sugar during subsequent pregnancy in a patient cannot be completely ruled out, although the proportion of this population is relatively low. Therefore, it has little effect on the result.

## Conclusion

This study demonstrated that pregnant women with diabetes had poorer sleep quality and a higher risk of developing OSAHS than pregnant women without diabetes. There may be some link between sleep quality and the onset of diabetic. Clinicians should pay more attention to sleep quality in high-risk pregnant women, with timely screening and interventions. This study lays the foundation for further studies and provides new ideas for the prevention and treatment of pregnant women with diabetes.

## Data Availability

All data generated or analyzed during this study are included in this published article.

## References

[1] Holmes VA, Young IS, Patterson CC, Pearson DW, Walker JD, Maresh MJ, McCance DR (2011). Diabetes, pre-eclampsia intervention Trial Study G: optimal glycemic control, pre-eclampsia, and gestational hypertension in women with type 1 diabetes in the diabetes and pre-eclampsia intervention trial. Diabetes Care.

[2] American Diabetes A (2020). 14. Management of diabetes in pregnancy: Standards of Medical Care in Diabetes-2020. Diabetes Care.

[3] Okun ML, Roberts JM, Marsland AL, Hall M (2009). How disturbed sleep may be a risk factor for adverse pregnancy outcomes. Obstet Gynecol Surv.

[4] Foley D, Ancoli-Israel S, Britz P, Walsh J (2004). Sleep disturbances and chronic disease in older adults: results of the 2003 National Sleep Foundation Sleep in America Survey. J Psychosom Res.

[5] Gottlieb DJ, Redline S, Nieto FJ, Baldwin CM, Newman AB, Resnick HE, Punjabi NM (2006). Association of usual sleep duration with hypertension: the Sleep Heart Health Study. Sleep.

[6] Prinz P (2004). Sleep, appetite, and obesity–what is the link?. PLoS Med.

[7] Doo H, Chun H, Doo M (2016). Associations of daily sleep duration and dietary macronutrient consumption with obesity and dyslipidemia in Koreans: a cross-sectional study. Med (Baltim).

[8] Itani O, Jike M, Watanabe N, Kaneita Y (2017). Short sleep duration and health outcomes: a systematic review, meta-analysis, and meta-regression. Sleep Med.

[9] Saadati F, Sehhatiei Shafaei F, Mirghafourvand M (2018). Sleep quality and its relationship with quality of life among high-risk pregnant women (gestational diabetes and hypertension). J maternal-fetal neonatal medicine: official J Eur Association Perinat Med Federation Asia Ocean Perinat Soc Int Soc Perinat Obstet.

[10] Wang H, Leng J, Li W, Wang L, Zhang C, Li W, Liu H, Zhang S, Chan J, Hu G (2017). Sleep duration and quality, and risk of gestational diabetes mellitus in pregnant chinese women. Diabet medicine: J Br Diabet Association.

[11] Wang Y, Huang W, O’Neil A, Lan Y, Aune D, Wang W, Yu C, Chen X (2020). Association between sleep duration and mortality risk among adults with type 2 diabetes: a prospective cohort study. Diabetologia.

[12] Wong PM, Manuck SB, DiNardo MM, Korytkowski M, Muldoon MF (2015). Shorter sleep duration is associated with decreased insulin sensitivity in healthy white men. Sleep.

[13] Patel SR, Malhotra A, Gottlieb DJ, White DP, Hu FB (2006). Correlates of long sleep duration. Sleep.

[14] Zheng R, Niu J, Wu S, Wang T, Wang S, Xu M, Chen Y, Dai M, Zhang D, Yu X (2021). Gender and age differences in the association between sleep characteristics and fasting glucose levels in chinese adults. Diabetes Metab.

[15] Basevi V, Di Mario S, Morciano C, Nonino F, Magrini N. Comment on: American Diabetes Association. Standards of medical care in diabetes–2011. Diabetes Care 2011;34(Suppl. 1):S11-S61. *Diabetes Care* 2011, 34(5):e53; author reply e54. 10.2337/dc11-0174 PMC3114493 21525493

[16] American Diabetes A (2014). Diagnosis and classification of diabetes mellitus. Diabetes Care.

[17] Consensus P, Metzger BE, Gabbe SG, Persson B, Buchanan TA, Catalano PA, Damm P, Dyer AR, Leiva A, International Association of D, Pregnancy Study Groups (2010). International association of diabetes and pregnancy study groups recommendations on the diagnosis and classification of hyperglycemia in pregnancy. Diabetes Care.

[18] Buysse DJ, Reynolds CF, Monk TH, Berman SR, Kupfer DJ (1989). The Pittsburgh Sleep Quality Index: a new instrument for psychiatric practice and research. Psychiatry Res.

[19] Tsai PS, Wang SY, Wang MY, Su CT, Yang TT, Huang CJ, Fang SC (2005). Psychometric evaluation of the chinese version of the Pittsburgh Sleep Quality Index (CPSQI) in primary insomnia and control subjects. Qual Life Res.

[20] Zhong QY, Gelaye B, Sanchez SE, Williams MA (2015). Psychometric Properties of the Pittsburgh Sleep Quality Index (PSQI) in a cohort of peruvian pregnant women. J Clin Sleep Med.

[21] Qiu C, Gelaye B, Zhong QY, Enquobahrie DA, Frederick IO, Williams MA (2016). Construct validity and factor structure of the Pittsburgh Sleep Quality Index among pregnant women in a Pacific-Northwest cohort. Sleep Breath.

[22] Chung F, Yegneswaran B, Liao P, Chung SA, Vairavanathan S, Islam S, Khajehdehi A, Shapiro CM (2008). STOP questionnaire: a tool to screen patients for obstructive sleep apnea. Anesthesiology.

[23] Sedov ID, Cameron EE, Madigan S, Tomfohr-Madsen LM (2018). Sleep quality during pregnancy: a meta-analysis. Sleep Med Rev.

[24] Silvestri R, Arico I (2019). Sleep disorders in pregnancy. Sleep Sci.

[25] Felder JN, Baer RJ, Rand L, Jelliffe-Pawlowski LL, Prather AA (2017). Sleep disorder diagnosis during pregnancy and risk of Preterm Birth. Obstet Gynecol.

[26] Tomfohr-Madsen L, Cameron EE, Dunkel Schetter C, Campbell T, O’Beirne M, Letourneau N, Giesbrecht GF (2019). Pregnancy anxiety and preterm birth: the moderating role of sleep. Health Psychol.

[27] Vizzini L, Popovic M, Zugna D, Vitiello B, Trevisan M, Pizzi C, Rusconi F, Gagliardi L, Merletti F, Richiardi L (2019). Maternal anxiety, depression and sleep disorders before and during pregnancy, and preschool ADHD symptoms in the NINFEA birth cohort study. Epidemiol Psychiatr Sci.

[28] Cai S, Tan S, Gluckman PD, Godfrey KM, Saw SM, Teoh OH, Chong YS, Meaney MJ, Kramer MS, Gooley JJ. Sleep quality and nocturnal sleep duration in pregnancy and risk of gestational diabetes Mellitus. Sleep 2017, 40(2). 10.1093/sleep/zsw058 28364489

[29] He J, Chen X, Wang Y, Liu Y, Bai J (2021). The experiences of pregnant women with gestational diabetes mellitus: a systematic review of qualitative evidence. Rev Endocr Metab Disord.

[30] Alwash SM, McIntyre HD, Mamun A (2021). The association of general obesity, central obesity and visceral body fat with the risk of gestational diabetes mellitus: evidence from a systematic review and meta-analysis. Obes Res Clin Pract.

[31] Reutrakul S, Anothaisintawee T, Herring SJ, Balserak BI, Marc I, Thakkinstian A (2018). Short sleep duration and hyperglycemia in pregnancy: aggregate and individual patient data meta-analysis. Sleep Med Rev.

[32] Xu YH, Shi L, Bao YP, Chen SJ, Shi J, Zhang RL, Lu L (2018). Association between sleep duration during pregnancy and gestational diabetes mellitus: a meta-analysis. Sleep Med.

[33] Rawal S, Hinkle SN, Zhu Y, Albert PS, Zhang C (2017). A longitudinal study of sleep duration in pregnancy and subsequent risk of gestational diabetes: findings from a prospective, multiracial cohort. Am J Obstet Gynecol.

[34] Cai S, Tan S, Gluckman PD, Godfrey KM, Saw SM, Teoh OH, Chong YS, Meaney MJ, Kramer MS, Gooley JJ et al. Sleep quality and nocturnal sleep duration in pregnancy and risk of gestational diabetes Mellitus. Sleep 2017, 40(2). 10.1093/sleep/zsw058 28364489

[35] Facco FL, Grobman WA, Reid KJ, Parker CB, Hunter SM, Silver RM, Basner RC, Saade GR, Pien GW, Manchanda S (2017). Objectively measured short sleep duration and later sleep midpoint in pregnancy are associated with a higher risk of gestational diabetes. Am J Obstet Gynecol.

[36] Zhong C, Chen R, Zhou X, Xu S, Li Q, Cui W, Wang W, Li X, Wu J, Liu C (2018). Poor sleep during early pregnancy increases subsequent risk of gestational diabetes mellitus. Sleep Med.

[37] Zhang C, Shen Y, Liping F, Ma J, Wang GF (2021). The role of dry mouth in screening sleep apnea. Postgrad Med J.

